# Use of Unidirectional Porous β-Tricalcium Phosphate in the Tibial Tunnel for Anterior Cruciate Ligament Reconstruction: A Case Series

**DOI:** 10.7759/cureus.58366

**Published:** 2024-04-16

**Authors:** Arata Watanabe, Naoya Kikuchi, Takumi Ichihara, Hiroshi Kumagai, Yu Taniguchi, Yuki Sato, Tomonori Kinugasa, Kotaro Ikeda, Masashi Yamazaki

**Affiliations:** 1 Department of Orthopaedic Surgery, Ichihara Hospital, Tsukuba, JPN; 2 Department of Orthopaedic Surgery, Institute of Medicine, University of Tsukuba, Tsukuba, JPN; 3 Department of Orthopaedic Surgery, Faculty of Medicine, University of Tsukuba, Tsukuba, JPN

**Keywords:** synthetic bone, bone remodeling, tibial tunnel, anterior cruciate ligament reconstruction, unidirectional porous β-tricalcium phosphate

## Abstract

Bone defects in the tibial tunnel for anterior cruciate ligament (ACL) reconstruction can cause adverse events. The unidirectional porous tricalcium β-phosphate (UDPTCP) has the potential to be used as a filling substitute for bone defects. In this case series, we present the first nine cases in which UDPTCP was used as a bone substitute in the tibial tunnel during ACL reconstruction. The patients comprised six males and three females, with an average age of 32 years (range: 16-50 years). A cylindrical UDPTCP measuring 10 x 20 mm was molded to fit the tibial tunnel and then implanted. At the one-year postoperative follow-up, none of the patients demonstrated any complications, and bone remodeling was observed on radiographs. Therefore, UDPTCP may provide a safe and reliable filling substitute for the tibial tunnel in ACL reconstruction.

## Introduction

Anterior cruciate ligament (ACL) reconstruction is a widely performed surgery for ACL injuries, yielding positive outcomes [1,2]. Despite these successes, postoperative complications such as infections, intra-articular bleeding, deep vein thrombosis, and pulmonary embolism can occur, with incidences ranging from 1% to 15% [3]. Among these, fractures on the femoral and tibial sides, often linked to weakened bones around the bone tunnels, are notable [4,5]. Specifically, fractures through the tibial tunnel have been documented in ACL reconstruction [6].

Prompt bone defect filling and regeneration in the tibial tunnel post-ACL reconstruction are vital to improve stability and reduce complication risks. Choosing materials that promote efficient bone regeneration in these defects is crucial for the procedure’s success.

Affinos® (Kuraray, Osaka, Japan) is an innovative, unidirectional porous β-tricalcium phosphate (UDPTCP) with a porosity of 57%. It has interconnected pores oriented in one direction, measuring from 25 to 300 μm. Its unique ability to balance bone formation and absorption, and its effective replacement by natural bone, have proven advantageous in various orthopedic procedures [7].

In this report, we describe our experience using UDPTCP in the tibial tunnel post-ACL reconstruction in nine patients.

## Materials and methods

Case summary

This study included nine patients who underwent either primary or revision ACL reconstruction. The cohort consisted of six males and three females, with an average age of 32 years (range: 16-50 years). The indications for surgery included five cases of primary reconstruction using the hamstring (semitendinosus and/or gracilis tendon) graft for single-bundle reconstruction and four cases of revision reconstruction utilizing quadriceps tendon-bone grafts. The diameter of the tibial tunnel was 8.5 mm in one case, 9 mm in five cases, 9.5 mm in two cases, and 10 mm in one case (Table 1).

**Table 1:** Summary of patients ACLR: anterior cruciate ligament reconstruction; HS: hamstring tendon graft; QTB: quadriceps-tendon bone graft

Case	Age	Sex	Surgery type	Graft type	Tibial tunnel diameter (mm)
1	49	Female	Primary ACLR	HS	10
2	19	Male	Revision ACLR	QTB	9
3	39	Male	Primary ACLR	HS	9
4	31	Male	Primary ACLR	HS	9
5	50	Female	Primary ACLR	HS	9.5
6	39	Male	Revision ACLR	QTB	9.5
7	17	Female	Revision ACLR	QTB	9
8	28	Male	Primary ACLR	QTB	8.5
9	16	Male	Revision ACLR	HS	9

Surgical procedures

Arthroscopic ACL reconstruction was performed utilizing semitendinosus and/or gracilis tendon for primary surgery and quadriceps tendon-bone grafts for revision surgery. The placement of the tibial tunnel was centralized within the ACL's anatomical footprint, while the femoral tunnel was situated posterior to the resident's ridge also centralized within the ACL footprint. The graft was inserted through the tibial tunnel, and the femoral side of the graft was secured using an adjustable loop button. On the tibial side, fixation was achieved with a spike-plate and a sutured screw at 20° knee flexion. During tibial fixation, the graft was manually tensioned to 30 N. Finally, a 10 x 20 mm cylindrical UDPTCP was molded to the dimensions of the tibial tunnel and subsequently implanted.

Outcome measurements

**Figure 1:**
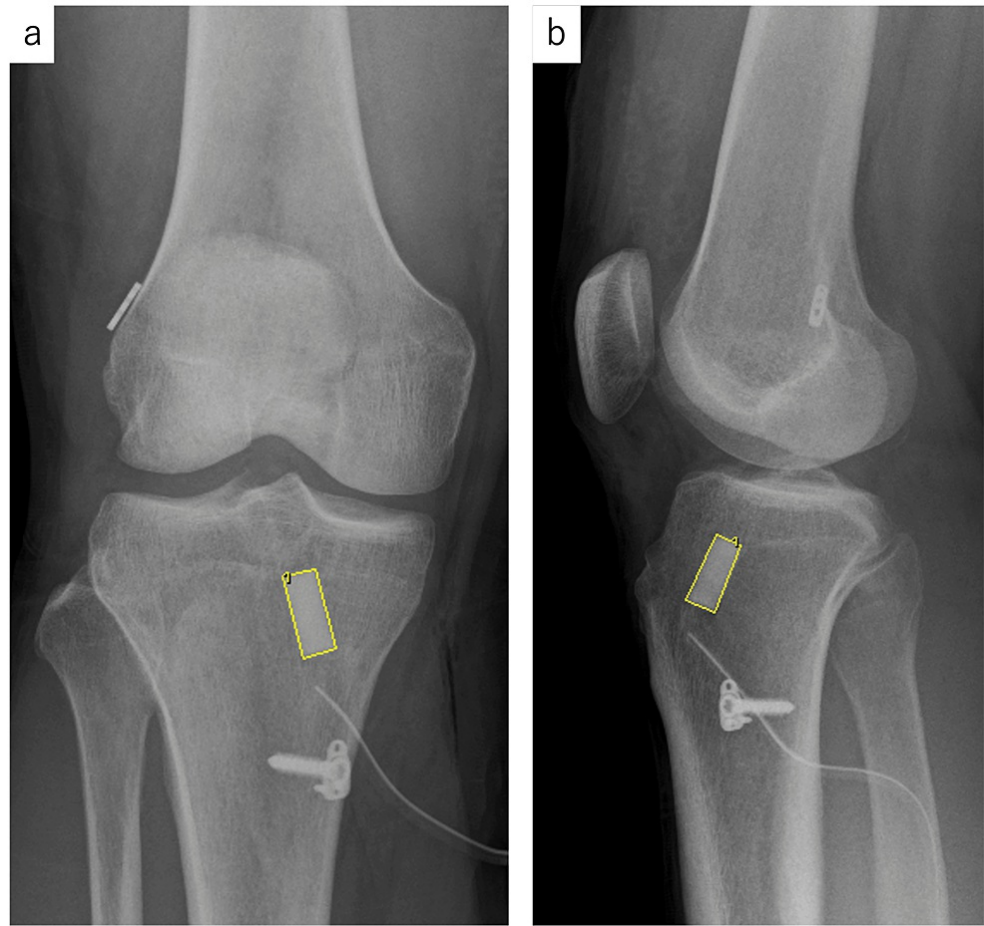
Measurement of the UDPTCP area (yellow rectangle) in (a) antero-posterior and (b) lateral views of radiograph

Image assessments were conducted using the picture archiving and communication system for assessing bone remodeling. The area occupied by UDPTCP within the tibial tunnel was quantified on anterior-posterior and lateral X-ray images both immediately after surgery and one year postoperatively (Figure 1). In addition, adverse events such as infections and allergies were investigated.

## Results

Comparative analysis of the UDPTCP area in X-ray, from immediately after the operation to one year later, demonstrated significant bone resorption and remodeling, indicating effective osteointegration (Table 2). None of the patients exhibited signs of infection, allergic reaction, or foreign body response. Here, we showed representative two cases.

**Table 2:** UDPTCP area immediately and one year postoperatively ^a^A paired t-test was used to compare the immediate and one-year postoperative UDPTCP area. DPTCP: unidirectional porous tricalcium β-phosphate

UDPTCP area (mm^2^)	Immediate postoperatively	One year postoperatively	p-value^a^
Anterior-posterior view	160.5 ± 34.9	73.6 ± 37.9	< 0.001
Lateral view	160.7 ± 19.0	93.0 ± 24.1	< 0.001

Case 4

**Figure 2:**
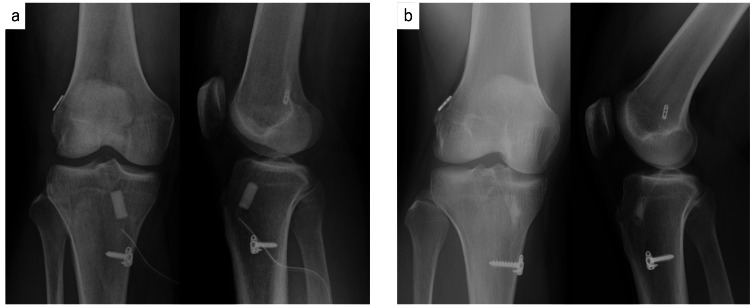
Antero-posterior and lateral views of radiographs at (a) immediate postoperative and (b) one year postoperative for case 4

Case 4 was a 31-year-old man who suffered an ACL tear while playing soccer. He was treated with conservative therapy, but continued to experience knee instability and underwent surgery one year and two months after the injury. The semitendinosus tendon was used as the graft tendon. Bone tunnels were created at anatomic locations in both the femur and tibia. The femoral side was fixed with adjustable-loop cortical button devices, and the tibial side was fixed with a spike plate and screws. The tibial tunnel was filled with 9 mm diameter UDPTCP. Postoperatively, the patient underwent rehabilitation under the supervision of a physical therapist and experienced no complications. One year after surgery, he was playing sports at the same level as before the injury. Plain radiographs showed that the immediate postoperative area was 176.6 mm² in the anterior-posterior view and 166.7 mm² in the lateral view; one year after surgery, this had shrunk to 51.4 mm² in the anterior-posterior view and 88.8 mm² in the lateral view, with clear bone remodeling observed (Figure 2).

Case 7

**Figure 3:**
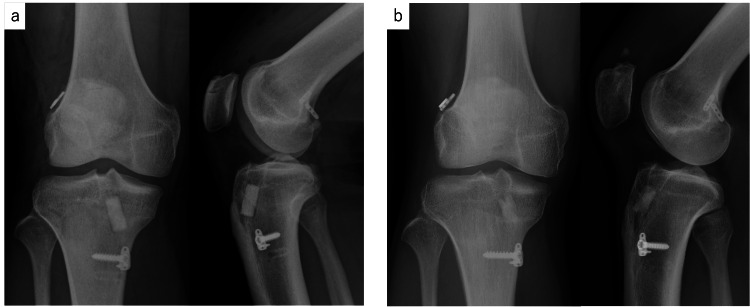
Antero-posterior and lateral views of radiographs at (a) immediate postoperative and (b) one year postoperative for case 7

Case 7 was a 17-year-old man who suffered an ACL tear while playing handball. He underwent ACL reconstruction using the semitendinosus tendon two weeks after the injury. Nine months after the initial surgery, he sustained an ACL re-injury while playing handball, for which he underwent reoperation one month later. The quadriceps bone tendon was used as the graft tendon. As the tunnel positions for the primary surgery were appropriate, the bone tunnels were created at anatomic locations in both the femur and tibia. The femoral side was fixed with adjustable-loop cortical button devices, and the tibial side was fixed with a spike plate and screws. The tibial tunnel was filled with 9 mm diameter UDPTCP. Postoperatively, the patient underwent rehabilitation under the supervision of a physical therapist and experienced no complications. Plain radiographs showed that the immediate postoperative area was 176.6 mm² in the anterior-posterior view and 166.7 mm² in the lateral view; one year after surgery, this had shrunk to 51.4 mm² in the anterior-posterior view and 88.8 mm² in the lateral view, with clear bone remodeling observed (Figure 3).

## Discussion

In our case series, using UDPTCP for bone grafting in the tibial tunnels during ACL reconstruction led to no complications and notable bone remodeling within one year, affirming its safety and efficacy.

Animal studies suggest that the oriented structure of UDPTCP aids rapid blood penetration via capillary action, facilitating early bone remodeling [8]. Despite its β-TCP composition, the porosity and structure of UDPTCP ensure balanced bone formation and resorption, promoting early remodeling for sustained bone stability and functional recovery [7]. Moreover, the application of UDPTCP extends beyond the tibia, with successful outcomes in spinal surgeries and filling bone defects post-tumor excision reported previously [9-12]. These cases highlight the bone resorption capacity of UDPTCP starting within three months, with almost complete remodeling in six months to one year. In the present case series, the area of UDPTCP was reduced at one year postoperatively, similar to its use in other sites.

To our knowledge, no prior studies have examined artificial bone use in the tibial tunnel for ACL reconstruction. Post ACL reconstruction, tibial plateau fractures typically result from tunnel enlargement or proximal tibia weakening, commonly occurring after one year [13-15]. While specific biomechanical studies are lacking, cortical defects from the tunnel likely act as stress risers, increasing fracture risk [16]. Further studies are warranted to determine whether it can reduce fractures and other adverse events.

Artificial bone usage could enhance early structural strength and mitigate fracture risks. A study contrasting spherical porous β-TCP and UDPTCP in the proximal tibia showed UDPTCP's superior early bone remodeling capability, without compromising strength [17-19]; even in ACL reconstruction, it is debatable which substitute is most appropriate. Multiple considerations such as strength, remodeling speed, and cost need to be considered.

This study has several limitations that must be acknowledged. Firstly, the data derived from our series have not undergone comparative analysis with other synthetic bone materials nor have they been thoroughly evaluated using computed tomography, which could provide further insights into the structural properties of the UDPTCP. Additionally, the potential synergistic effects of biological factors, such as platelet-rich plasma [20], on the bone remodeling process facilitated by UDPTCP have not been explored. These factors could play a critical role in enhancing the efficacy of UDPTCP in the context of bone regeneration and need further investigation. Despite these limitations, it is worthwhile to be the first to demonstrate the bone remodeling and safety of UDPTCP implanted in the tibial tunnel of ACL reconstruction.

## Conclusions

At one year postoperatively, the area of UDPTCP was smaller than immediately after surgery. Furthermore, no adverse events were observed in all patients.

In our case series, the findings show that UDPTCP could be a safe and reliable filling material with good biocompatibility and rapid absorption characteristics for filling the tibial tunnel during ACL reconstruction.
